# Two‐Scope Technique for Tracheal Epithelial‐Myoepithelial Carcinoma‐A Rare Case Report and Review

**DOI:** 10.1002/rcr2.70333

**Published:** 2025-09-07

**Authors:** Saurabh Karmakar, Vinay Venugopal, Abdul Raouf Wani, Gaurav Kumar Singh, Debapriya Maji, Alpana Srivastava

**Affiliations:** ^1^ Department of Pulmonary Medicine All India Institute of Medical Sciences (AIIMS) Patna India; ^2^ Core Diagnostics New Delhi India

**Keywords:** central airway lesion, pulmonary epithelial‐myoepithelial carcinoma, rare salivary gland‐type neoplasm, two scope technique

## Abstract

Epithelial myoepithelial carcinoma (EMC) is an exceptionally uncommon salivary gland type tumour of the lung accounting for only 0.1% to 1% of all primary pulmonary malignancies. We describe a 40‐year‐old man presenting with hemoptysis, in whom computed tomography (CT) of the chest revealed an endoluminal mass in the upper trachea. The initial biopsy suggested adenoid cystic carcinoma. Following complete removal using two‐scope flexible bronchoscopic technique combining tumour snaring with cryoprobe‐assisted retrieval, the final diagnosis of primary tracheal EMC was confirmed on histopathology and immunohistochemistry. This case not only contributes to the limited reports of tracheal EMC but also illustrates that the two‐scope method may be a practical alternative to rigid bronchoscopy, especially in resource‐limited or complex airway situations.

## Graphical abstract

Our case describes a rare tumour, tracheal epithelial‐myoepithelial carcinoma, that was successfully removed using a two‐scope technique (novel technique for tumour resection). The diagnosis was confirmed by comprehensive histopathological and immunohistochemical examination. At 6 months of follow‐up, the patient remains well and free of recurrence, suggesting that this approach can serve as a safe and practical alternative in selected cases.

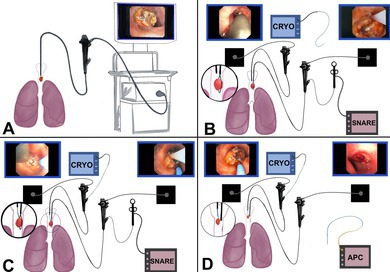

## 1 Introduction

Salivary gland tumours (SGTs) are uncommon neoplasms contributing to < 1% to 5% of all head and neck malignancies [1]. The salivary gland‐type tumours (SGTTs) of the lung are categorised into seven distinct neoplasms based on the 2021 World Health Organization (WHO) classification: adenoid cystic carcinoma, mucoepidermoid carcinoma, hyalinizing clear cell carcinoma, pleomorphic adenoma, epithelial‐myoepithelial carcinoma, myoepithelioma, and myoepithelial carcinoma [2]. Pulmonary epithelial‐myoepithelial carcinoma (P‐EMC) is exceptionally rare, comprising only 0.1% to 1% of primary lung cancers [3]. Owing to its rarity and uncertain malignant potential, standardised management strategies are not well established. To the best of our knowledge, this is the first documented case of a central airway mass lesion successfully resected using the two‐scope technique with minimal complications.

## 2 Case Report

**FIGURE 1:**
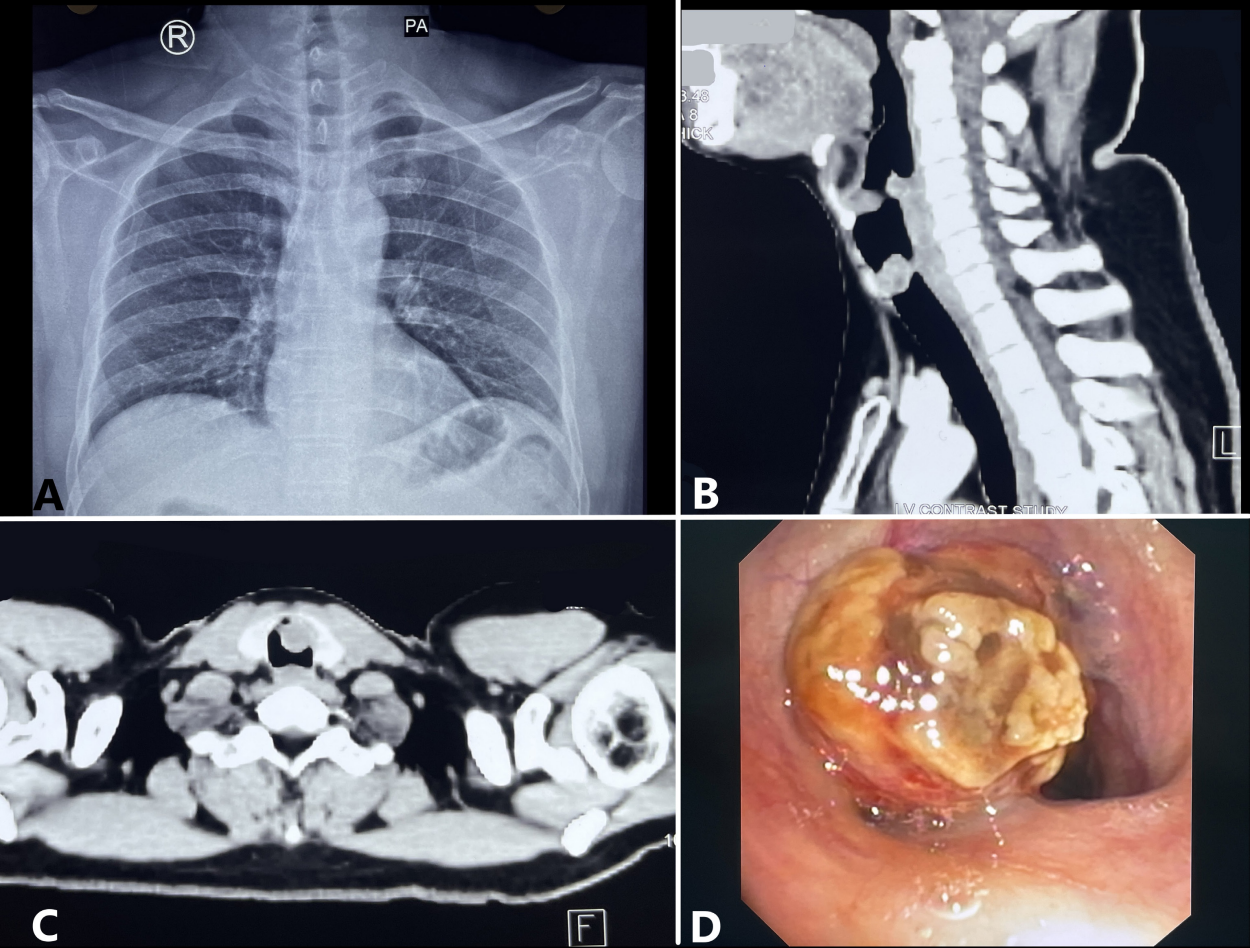
Chest X‐ray PA view reveals no obvious abnormality (A); CECT Neck reveals an endoluminal polypoidal soft tissue mass in the upper trachea measuring 1.7 × 1.8 cm at C4‐C5 level (B) (Sagittal section) and (C) (Axial section). Flexible bronchoscopy showing mass in the sub‐glottic region occluding half of tracheal lumen (D).

A 40‐year‐old man presented with 2 weeks of hemoptysis without other symptoms like shortness of breath, difficulty in swallowing, hoarseness, fatigue, and weight loss. He had no history of asthma, prior hospitalisation, trauma, or substance abuse. Family history was not significant. Physical examination was unremarkable. There was no pallor, icterus, cyanosis, or palpable lymph node. Respiratory system examination revealed bilateral wheeze. Rest all other systems were unremarkable. Laboratory investigations showed normal haematological parameters. Chest X‐ray (PA view) is shown in Figure 1A. A contrast‐enhanced computed tomography (CECT) of the chest revealed a 1.7 × 1.8 cm heterogeneously enhancing endoluminal polypoidal soft tissue mass in the upper trachea (C4‐C5 level) (Figure 1B,C). Spirometry revealed a predicted forced Expiratory Volume (FEV1) of 109% (3.13 L), predicted forced vital capacity (FVC) of 106% (3.82 L) and an FEV1/FVC ratio of 82%. Fibreoptic bronchoscopy (FOB) showed a sub‐glottic mass obstructing approximately half of the lumen (Figure 1D). Initial histopathological examination of the biopsy indicated adenoid cystic carcinoma.

Given the clinical scenario and patient's decline for surgery, we planned endoscopic removal of the tumour with prior consideration of super‐selective embolization. However, angiography did not show any significant arterial supply to the mass. The two‐scope flexible bronchoscopy technique was chosen for tumour resection due to the following reasons: non‐availability of a rigid bronchoscope, inability to secure the airway with an endotracheal (ET) tube as the mass was located at the subglottic region, and only a single therapeutic scope can be passed through the laryngeal mask airway (LMA) which carries a risk of tumour migration after resection.

The procedure required a team of four doctors (two for scopes and two for cryoprobe/snare) and two staff (One for administering IV sedatives and other to handle the sample). The procedure was explained to the patient and informed consent was obtained.

The entire procedure was performed under local anaesthesia with conscious sedation. Topical anaesthesia was administered with four sprays of 10% lignocaine to the pharynx. Sedation was achieved using intravenous (IV) midazolam (2 mg) and fentanyl (50 mcg). Low‐flow supplemental oxygen (2–3 L/min) was delivered via nasal cannula. Continuous monitoring of oxygen saturation, heart rate, and blood pressure was performed throughout the procedure.

**FIGURE 2:**
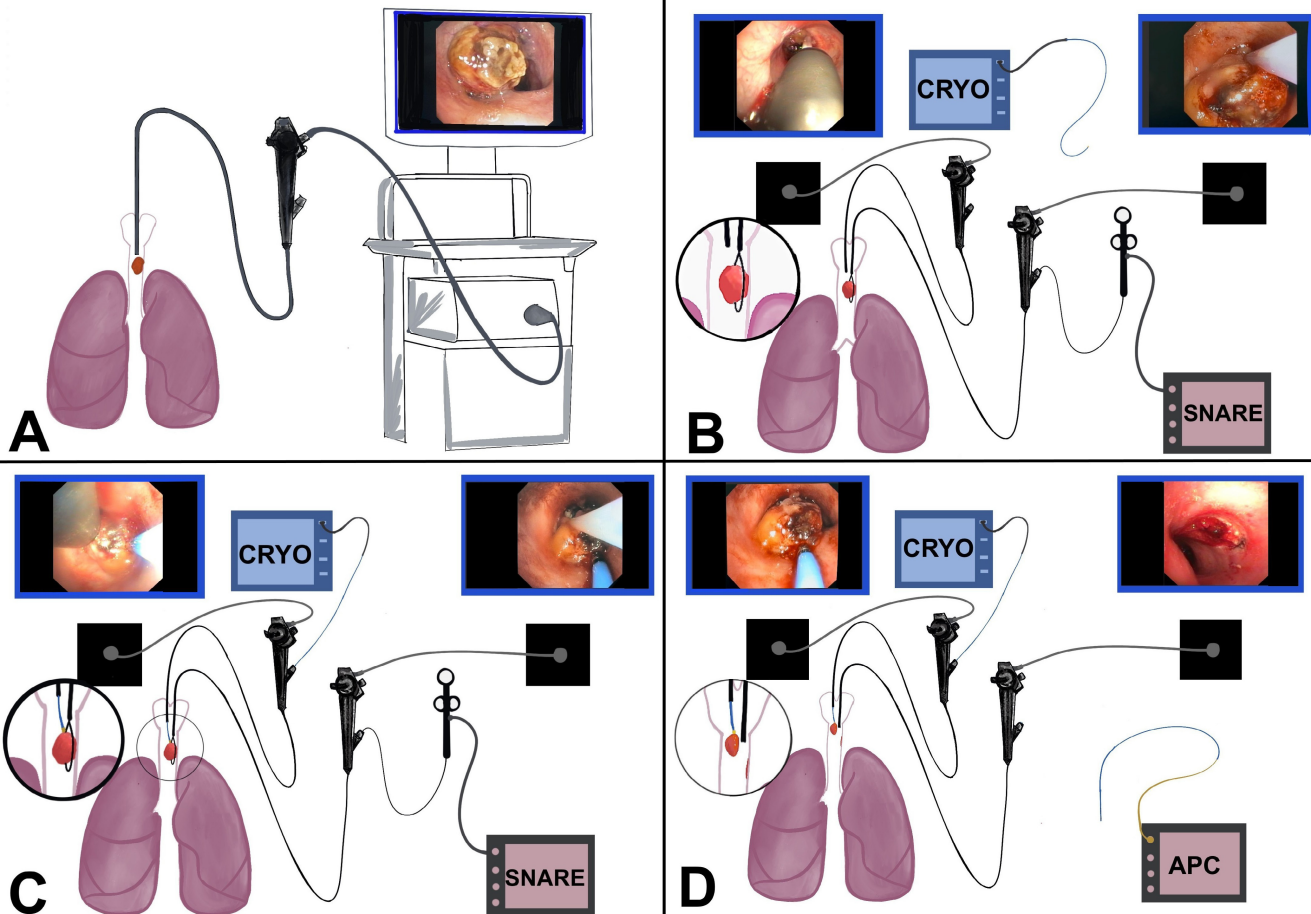
Demonstration of the two‐scope technique using two adult conventional bronchoscopes (6 mm outer diameter). First bronchoscope is used to snare the mass (A, B), while the second bronchoscope is used to extract the resected mass with a 2.4 mm cryoprobe (C, D).

Once adequate sedation was confirmed, a bite block was positioned in the mouth. Adult therapeutic flexible video bronchoscope (Olympus BF‐1TQ170, Olympus Corporation, Japan; channel diameter 2.8 mm, outer diameter 6.0 mm) was introduced orally to visualise the tracheal mass [Figure 2A]. The “spray‐as‐you‐go” method with 1% lignocaine was applied for anaesthetising the vocal cord and trachea.

Using the first scope, the tumour base was looped with a snare [Figure 2B right]. Simultaneously, a second flexible bronchoscope (Olympus BF‐1 T150 (adult), Olympus Corporation, Japan; channel diameter 2.8 mm, outer diameter 6.0 mm) was introduced through the same bite block visualising ensnaring the mass [Figure 2B left]. The cryoprobe (2.4 mm flexible cryoprobe; ERBOKRYO CA, ERBE, Germany) was introduced via the second bronchoscope [Figure 2C right]. It was activated simultaneously during tumour snaring [Figure 2C left], allowing en bloc extraction of the mass [Figure 2D left]. The first scope remained in the trachea to monitor bleeding [Figure 2D right].

The base of the resection site was cauterised using argon plasma coagulation through the first scope, effectively controlling the minimal bleeding and reducing the risk of tumour re‐growth. After resection, the tracheal lumen was inspected to ensure complete clearance and haemostasis before concluding the procedure. The total duration of the procedure was 15 min.

**FIGURE 3:**
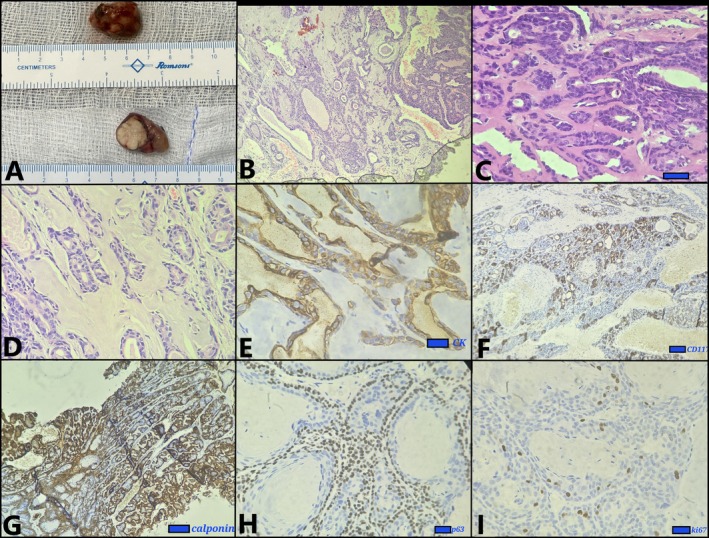
Gross image of the resected tracheal mass demonstrating the endoluminal surface and cut section (A); Histopathological examination shows tumour in the lamina propria composed of bi‐layered tubular structures with inner ductal cells and outer round to polygonal myoepithelial cells (B–D). Immunohistochemistry (E–I) reveals luminal cells positive for cytokeratin (CK) (E), and CD117 (F); abluminal cells were positive for calponin (G) and p63 (H). The Ki‐67 proliferation index is ~10% (I).

The pathological evaluation of the resected mass [Figure 3A] showed a biphasic tumour composed of inner ductal epithelial cells surrounded by polygonal myoepithelial cells [Figure 3B–D]. Immunohistochemistry (IHC) demonstrated luminal cell positivity for cytokeratin (CK), CK7, CD117, whereas the abluminal cells were positive for vimentin, calponin, p63, and focal S‐100 [Figure 3E–H]. No nuclear beta‐catenin staining was seen, and the Ki‐67 proliferation index was ~10% (Figure 3I). These findings confirmed the diagnosis of primary T‐EMC. Whole‐body Fluorodeoxyglucose‐Positron Emission Tomography/Computed Tomography (FDG‐PET/CT) showed no residual or metastatic disease, and the patient remains recurrence‐free at 6 months of follow‐up.

## 3 Discussion

Epithelial‐myoepithelial carcinoma (EMC) is an uncommon salivary‐type tumour with low malignant potential, accounting for < 1% of all salivary gland neoplasms. Pulmonary and tracheal EMCs are even rarer and usually follow an indolent course. It usually arises from the submucosal glands of the airway or occasionally from lung parenchyma [3, 4]. Historically, EMC was described under several different names including adeno‐myoepithelioma, clear cell adenoma, tubular solid adenoma, and clear cell carcinoma, reflecting its varied histopathologic appearance in early literature (1956). The term “epithelial‐myoepithelial carcinoma” was formally introduced by *Donath* et al. in 1972 in a case of clear cell carcinoma of the salivary gland [5]. T‐EMC is extremely rare; fewer than five are reported, including those by *Konoglou* et al. in 2013 [6], *Song* et al. in 2014 [7], *Huang* et al. in 2021 [8], along with the current case summarised in Table 1.

**TABLE 1:** Characteristics of reported cases of Tracheal Epithelial‐myoepithelial carcinoma (T‐EMC).

Ref	Year	Author	Age/sex	Size (cm)	Smoking status	Location	Luminal cell IHC	Abluminal cell IHC	Ki‐67	Treatment	Metastasis	Follow‐up (months)
[6]	2013	Konoglou et al.	34 yrs/M	1.15	+	Left lateral wall of trachea	CK7 (+); CK8/18 (+)	S‐100 (+); SMA (+); and P63 (+)	NA	Resection of five tracheal rings	NA	FOD: 24 months
[7]	2014	Song et al.	63 yrs/F	2	−	Distal trachea	CK (+); TTF‐1 (+)	S‐100 (+); SMA (+); and P27 (+)	NA	Endobronchial excision	No	FOD: 10 months
[8]	2021	Huang et al. Case 1	70 yrs/M	5	+	Anterior wall of upper trachea	CK7 (+); CD117 (+); CK8 (+); and TTF (−)	S‐100 (+); SMA (+); CK5/6 (+); P63 (+); and P40 (+)	10%	Electrocautery snare, biopsy forceps and aspirator	NA	NA
	2025	Present case	40/M	1.8	−	Anterior wall of upper trachea	CK (+); CD117 (+); CK7 (+)	Vim (+); Calponin (+); p63 (+); and S‐100 (+)	10%	Electrocautery snare and cryoextraction	No	6 months

Abbreviations: CK, cytokeratin; F, female; FOD, free of disease; M, male; NA, not available; SMA, smooth muscle actin; vim, vimentin.

Pulmonary EMC is found to be more common in females, especially in middle‐aged and elderly women. The usual location is endobronchial, and a common clinical feature is that it is secondary to bronchial obstruction [3]. T‐EMC has a male predominance in three out of the four cases, with an age range of 34 to 70 years. The smoking history was variable; two patients were smokers, while the others had no such history. All tumours were located on the anterior or lateral wall of the trachea and presented with symptoms such as hemoptysis, airway obstruction, wheezing, and audible stridor. The tumour size ranged from 1.15 cm to 5 cm, with the lesion in our case measuring 1.7 × 1.8 cm. Clinically, symptoms such as airway obstruction, wheeze, stridor, and hemoptysis are common and can mimic asthma, which can delay the diagnosis. CT imaging is commonly used for the initial evaluation of T‐EMC, which often reveals a polypoidal endoluminal mass [6, 7, 8].

Imaging typically reveals a polypoidal endoluminal mass within the trachea, but a definitive diagnosis requires microscopic evaluation in combination with IHC. Three histologic variants of P‐EMC are recognised: a biphasic ductal‐myoepithelial pattern resembling salivary gland tumours, a predominantly solid form made up of spindle or polygonal myoepithelial cells within a myxoid stroma, and a myoepithelial‐rich subtype showing marked nuclear atypia in a highly myxoid background. Histopathology in our case demonstrated a biphasic pattern composed of duct‐forming epithelial cells surrounded by polygonal myoepithelial cells. On IHC, the inner luminal epithelial cells usually show positivity for markers like CK, CK7, CK8/18, CK903, CD117, EMA (epithelial membrane antigen), SPA (surfactant proteins A), and TTF‐1 (thyroid transcription factor‐1), P53, S‐100, CAM5.2, AE1/AE3, MNF‐116, and CD10. The outer abluminal myoepithelial cells stain positive for S‐100, SMA (smooth muscle actin), vimentin, calponin, P63, P40, P27, HHF‐35, GFAP (glial fibrillary acidic protein), and actin. The Ki‐67 proliferation index was approximately 10%, indicating low proliferative activity [3, 6, 7, 8]. This dual immunoprofile confirms the diagnosis of EMC and aids in distinguishing it from other salivary gland‐type tumours.

P‐EMC can mimic a range of pulmonary neoplasms which include pulmonary blastoma, carcinosarcoma, cartilaginous hamartoma, adenoid cystic carcinoma, primary pulmonary clear cell tumours, metastatic renal or thyroid clear cell carcinomas, pulmonary adenosquamous carcinoma, and glandular carcinoid tumours [3, 9]. The key distinguishing feature is the prominent myoepithelial component with a largely non‐invasive growth pattern [3]. In our case, the initial biopsy suggested ACC; but the final diagnosis was revised to EMC with extended immunophenotyping.

Complete **s**urgical excision remains to be the primary treatment for EMC, while endoscopic resection can be considered carefully in selected patients [3]. Varied treatments have been reported for T‐EMC; *Konoglou* et al. performed resection of five tracheal rings [6], whereas *Song* et al. [7] and *Huang* et al. [8] used electrocautery snaring, forceps‐based endobronchial excision, and aspirator. Chemotherapy and radiotherapy are rarely used and reserved for palliation in unresectable, locally recurrent, or metastatic disease. At present, the National Comprehensive Cancer Network (NCCN) does not provide a specific chemotherapy protocol. For advanced SGTTs, chemotherapy may be administered as a single agent or in combination including cisplatin, doxorubicin, cyclophosphamide, mitoxantrone, vinorelbine, and 5‐fluorouracil [3, 10]. Reports by *Pierard* et al. and *Yamazaki* et al. describe metastatic EMC cases achieving disease control with systemic therapy after initial treatment failure. Regimens included paclitaxel plus cyclophosphamide or cisplatin, docetaxel, and 5‐fluorouracil, which resulted in clinical improvement and stabilisation of lung lesions [11, 12]. Management is tailored according to individual risk factors such as the patient's overall status, tumour size, margin status, and the presence of lymph node involvement [3]. The American Society of Clinical Oncology (ASCO) recommends baseline post‐treatment imaging, preferably contrast‐enhanced CT or MRI, with or without FDG‐PET/CT around 3 months after completion of therapy [1].

*Chen* et al. reported multiple genetic alterations in a P‐EMC case including an HRAS Q61R mutation [13]. In salivary gland EMCs, HRAS mutations are common and often accompanied by alterations in PIK3CA and AKT1 genes [14]. Further research is needed to clarify the diagnostic, prognostic, and therapeutic implications of these mutations as no targeted therapy is currently available. Though P‐EMC is a low‐grade malignancy, it can rarely metastasize to bone, mediastinal lymph nodes, or lung [3]; however, all reported T‐EMC cases have no metastases suggesting a good prognosis [6, 7, 8]. Across all reported cases of T‐EMC, FDG‐PET/CT imaging has shown no evidence of local or distant metastasis. Reported cases remained disease‐free for 6 to 244 months, including our patient who is recurrence‐free at 6 months [6, 7, 8]. The low Ki‐67 index, absence of nodal involvement, and lack of invasive features all contribute to the favourable outcome. Our patient underwent successful bronchoscopic resection and remains recurrence‐free at 6 months.

Two‐scope technique has been described in the literature for obtaining transbronchial lung cryobiopsy in interstitial lung disease by *Sriprasart* et al. [15] and *Chawla* et al. [16], for sampling peripheral pulmonary lesion (PPL) by *Nakai* et al. [17], for performing cryobiopsy in PPLs using modified technique by *Kho* et al. [18], and for managing massive hemoptysis by *Baba* et al. [19]. In the current case, we employ two‐scope method for airway tumour resection using dual flexible conventional adult therapeutic bronchoscopes simultaneously: one scope is used to snare and resect the tumour, while the other retrieves the snared mass with a cryoprobe concurrently depicted in Figure 2. There was no lag time between two scopes in our case.

Laser ablation, particularly Nd: YAG, provides rapid, precise debulking with excellent haemostatic control in obstructive or vascular lesions. However, its use is limited by shallow tissue penetration (~10 mm), the need for specialised equipment and expertise, and the poor quality of tissue samples for histopathology. Rigid bronchoscopy is usually preferred to clear large debris, though flexible bronchoscopy can be used for distal lesions. In contrast, snare resection allows en bloc removal with histological confirmation, though the bleeding risk is higher in vascular tumours and its utility is limited in broad‐based lesions [20, 21].

The two‐scope approach allows en bloc tumour resection while preserving tissue architecture. It uses standard bronchoscopic tools (snare, cryoprobe, APC) and offers dual visualisation for added safety. This makes it a feasible, minimally invasive alternative for selected proximal airway lesions, reducing the risk of migration, particularly where rigid bronchoscopy or laser is unavailable. However, this technique is technically demanding, requires two bronchoscopes and a coordinated team, and may be less effective for bulky or highly vascular lesions. To our knowledge, this is the first reported case of central airway tumour resection using the two‐scope technique.

## Author Contributions

S.K. and V. V.: Planning and supervision; V. V., A.R.W., G.K.S., D.M.: Involved in patient's care, bronchoscopic procedure, and follow‐up; A.S.: Histopathology and IHC interpretation; V. V.: drafted the manuscript; while all authors reviewed, refined, and approved the final version together.

## Consent

The authors declare that written informed consent was obtained for the publication of this manuscript and accompanying images and attest that the form used to obtain consent from the patient complies with the Journal requirements as outlined in the author guidelines.

## Conflicts of Interest

The authors declare no conflicts of interest.

## Data Availability

The data that support the findings of this study are available from the corresponding author upon reasonable request.

## References

[1] J. L. Geiger , N. Ismaila , B. Beadle , et al., “Management of Salivary Gland Malignancy: ASCO Guideline,” Journal of Clinical Oncology 39 (2021): 1909–1941, 10.1200/JCO.21.00449. 33900808

[2] A. G. Nicholson , M. S. Tsao , M. B. Beasley , et al., “The 2021 WHO Classification of Lung Tumors: Impact of Advances Since 2015,” Journal of Thoracic Oncology 17, no. 3 (2022): 362–387. 34808341 10.1016/j.jtho.2021.11.003

[3] L. Chen , Y. Fan , and H. Lu , “Pulmonary Epithelial‐Myoepithelial Carcinoma,” Journal of Oncology 2022 (2022): 4559550. 36268279 10.1155/2022/4559550 PMC9578788

[4] S. H. Cho , S. D. Park , T. Y. Ko , H. Y. Lee , and J. I. Kim , “Primary Epithelial Myoepithelial Lung Carcinoma,” Korean Journal of Thoracic and Cardiovascular Surgery 47, no. 1 (2014): 59–62, 10.5090/kjtcs.2014.47.1.59. 24570870 PMC3928267

[5] K. Donath , G. Seifert , and R. Schmitz , “Diagnosis and Ultrastructure of the Tubular Carcinoma of Salivary Gland Ducts. Epithelial‐Myoepithelial Carcinoma of the Intercalated Ducts,” Virchows Archiv. A: Pathology. Pathologische Anatomie 356, no. 1 (1972): 16–31. 4340536

[6] M. Konoglou , A. Cheva , P. Zarogoulidis , et al., “Epithelial‐Myoepithelial Carcinoma of the Trachea—A Rare Entity Case Report,” Journal of Thoracic Disease 6, no. Suppl 1 (2014): S194–S199. 24672694 10.3978/j.issn.2072-1439.2013.11.17 PMC3966153

[7] D. H. Song , I. H. Choi , S. Y. Ha , et al., “Epithelial–Myoepthelial Carcinoma of the Tracheobronchial Tree: The Prognostic Role of Myoepithelial Cells,” Lung Cancer 83, no. 3 (2014): 416–419. 24485468 10.1016/j.lungcan.2014.01.005

[8] H. C. Huang , L. Zhao , X. h. Cao , G. Meng , Y. j. Wang , and M. Wu , “Primary Salivary Gland Tumors of the Lung: Two Cases Date Report and Literature Review,” Respiratory Medicine Case Reports 32 (2020): 101333. 33457200 10.1016/j.rmcr.2020.101333 PMC7797914

[9] R. W. Wilson and C. A. Moran , “Epithelial‐Myoepithelial Carcinoma of the Lung: Immunohistochemical and Ultrastructural Observations and Review of the Literature,” Human Pathology 28, no. 5 (1997): 631–635. 9158714 10.1016/s0046-8177(97)90088-5

[10] X. Wang , Y. Luo , M. Li , H. Yan , M. Sun , and T. Fan , “Management of Salivary Gland Carcinomas ‐ a Review,” Oncotarget 8, no. 3 (2016): 3946–3956. 10.18632/oncotarget.13952 PMC5354805 27992367

[11] S. Pierard , V. Gregoire , B. Weynand , and J. P. Machiels , “Epithelial–Myoepithelial Carcinoma of the Submandibular Gland With Symptomatic Lung Metastases Treated With Chemotherapy,” European Archives of Oto‐Rhino‐Laryngology 263, no. 12 (2006): 1158–1160. 16896751 10.1007/s00405-006-0125-8

[12] H. Yamazaki , Y. Ota , T. Aoki , and A. Kaneko , “Lung Metastases of Epithelial‐Myoepithelial Carcinoma of the Parotid Gland Successfully Treated With Chemotherapy: A Case Report,” Journal of Oral and Maxillofacial Surgery 71, no. 1 (2013): 220–226. 22695014 10.1016/j.joms.2012.03.031

[13] L. Chen , Q. Li , G. Fu , and M. Ge , “A Rare Case of Pulmonary Epithelial‐Myoepithelial Carcinoma: Case Report and Literature Review,” Zhongguo Fei Ai Za Zhi 23, no. 2 (2020): 127–132. 32093457 10.3779/j.issn.1009-3419.2020.02.08 PMC7049792

[14] M. Urano , M. Nakaguro , Y. Yamamoto , et al., “Diagnostic Significance of HRAS Mutations in Epithelial‐Myoepithelial Carcinomas Exhibiting a Broad Histopathologic Spectrum,” American Journal of Surgical Pathology 43, no. 7 (2019): 984–994. 30994537 10.1097/PAS.0000000000001258

[15] T. Sriprasart , A. Aragaki , R. Baughman , et al., “A Single US Center Experience of Transbronchial Lung Cryobiopsy for Diagnosing Interstitial Lung Disease With a 2‐Scope Technique,” Journal of Bronchology & Interventional Pulmonology 24, no. 2 (2017): 131–135. 28323726 10.1097/LBR.0000000000000366 PMC5367496

[16] R. K. Chawla, Sr. , R. Dhar, Sr. , A. Madan , et al., “Transbronchial Lung Cryobiopsy by Twin Bronchoscopes (Kissing Technique),” Indian Journal of Tuberculosis 68, no. 1 (2021): 16–19, 10.1016/j.ijtb.2020.10.010. 33641842

[17] T. Nakai , T. Watanabe , Y. Kaimi , et al., “Safety Profile and Risk Factors for Bleeding in Transbronchial Cryobiopsy Using a Two‐Scope Technique for Peripheral Pulmonary Lesions,” BMC Pulmonary Medicine 22, no. 1 (2022): 1–11. 35000601 10.1186/s12890-021-01817-8 PMC8744348

[18] S. S. Kho , C. S. Chai , and A. M. Ismail , “Modified Two‐Scope Technique for Transbronchial Lung Cryobiopsy of Peripheral Pulmonary Lesions,” Respirology Case Reports 12, no. 8 (2024): e01450. 39130086 10.1002/rcr2.1450 PMC11317175

[19] T. Baba , T. Ito , Y. Sato , et al., “Bronchial Occlusion With Endobronchial Watanabe Spigots Using a Two‐Scope Technique for Massive Haemoptysis,” Respirology Case Reports 12, no. 6 (2024): e01405. 38868562 10.1002/rcr2.1405 PMC11167016

[20] V. G. Pasricha , D. M. DiBardino , and K. C. Ma , “Management of Malignant Central Airway Obstruction,” Shanghai Chest 4 (2020): 26, 10.21037/shc.2020.01.02.

[21] P. D. Mitchell and M. P. Kennedy , “Bronchoscopic Management of Malignant Airway Obstruction,” Advances in Therapy 31, no. 5 (2014): 512–538. 24849167 10.1007/s12325-014-0122-z

